# The role of patients in the governance of a sustainable healthcare system: A scoping review

**DOI:** 10.1371/journal.pone.0271122

**Published:** 2022-07-13

**Authors:** Monica Aggarwal, Sukhraj Gill, Adeel Siddiquei, Kristina Kokorelias, Giulio DiDiodato

**Affiliations:** 1 Dalla Lana School of Public Health, University of Toronto, Toronto, Ontario, Canada; 2 Geisinger Medical Center, School of Medicine, Danville, Pennsylvania, United States of America; 3 North York General Hospital, General Assessment and Wellness Centre, Toronto, Ontario, Canada; 4 St John’s Rehab Program, Sunnybrook Research Institute, Sunnybrook Health Sciences Centre, Toronto, Ontario, Canada; 5 Department of Critical Care Medicine, Royal Victoria Regional Health Centre, Barrie, Ontario, Canada; La Trobe University - Melbourne Campus: La Trobe University, AUSTRALIA

## Abstract

Patients, healthcare providers and insurers need a governance framework to establish the ‘rules of use’ to deliver more responsible use of services. The objective of this review was to provide an overview of frameworks and analyze the definitions of patient accountability to identify themes and potential gaps in the literature. Fifteen bibliographic databases were searched until July 2021. This included: MEDLINE, EMBASE, CINAHL, PsycINFO, SPORTDiscus, Allied and Complementary Medicine Database, Web of Science, HealthSTAR, Scopus, ABI/INFORM Global, Cochrane Library, ERIC, International Bibliography of the Social Sciences, Sociological Abstracts, Worldwide Political Science Abstracts and International Political Science Abstracts. Searches were also completed in Google Scholar. Inclusion criteria included articles focused on accountability of patients, and exclusions included articles that were not available, not written in English, with missing information, and commentaries or editorials. In total, 85530 unique abstracts were identified, and 27 articles were included based on the inclusion criteria. The results showed that patient accountability is rarely used and poorly defined. Most studies focused on what patients should be held to account for and agreed that patients should be responsible for behaviours that may contribute to adverse health outcomes. Some studies promoted a punitive approach as a mechanism of enforcement. Most studies argued for positive incentives or written agreements and contracts. While many studies recognized the value of patient accountability frameworks, there was a concern that these frameworks could further exacerbate existing socioeconomic disparities and contribute to poor health-related behaviours and outcomes (e.g., stigmatizing marginalized groups). Shared models of accountability between patients and healthcare providers or patients and communities were preferred. Before committing to a patient accountability framework for improving patient health and sustaining a healthcare system, the concept must be acceptable and reasonable to patients, providers, and society as a whole.

## Introduction

Low-value care, defined as “patient care that provides no net benefit in specific clinical scenarios”, has become a concern at the global level [1 p. 333]. Low-value care is associated with harmful patient outcomes, wasteful spending and increased cost of care [2]. Few explorations of the cost of low-value care have been noted. In the United States, low-value care has been associated with cost-effectiveness ratios greater than $100 000 to $150 000 USD per quality-adjusted life-year (QALY) [3]. In Canada, the Canadian Institute for Health Information (2016) estimated that $228.1 billion ($6299 per Canadian) was spent on healthcare in Canada [1]. Of these costs, the public sector’s share of total healthcare spending (69.8 percent) was $159.1 billion. In 2016, this added up to approximately $148.3 billion (about $4,095 per person) of expenditures by provinces and territories [4]. From 2001, the costs have increased by 116.4 percent [5]. A recent report also found up to 30% of selected medical tests, treatments and procedures in Canada are potentially unnecessary and that Canadians have more than one million potentially unnecessary medical tests and treatments every year [6]. This pandemic of low-value care affects the sustainability of publicly funded healthcare systems globally, requiring patients, healthcare providers and policymakers to cooperate in finding socially and politically acceptable solutions [7–9].

The principles of the Canada Health Act stipulate that reasonable access to publicly administered insured health services must be comprehensively and universally guaranteed for all Canadians regardless of their provincial residence [10]. Less clear is what should the insured services be and what is reasonable? On two separate occasions, the Supreme Court of Canada has had to render decisions on these issues [11, 12]. Both times, the Court has ruled against the healthcare providers’ definitions of *reasonable* insured services, suggesting Canadians may view the healthcare system as an infinite resource instead of a common pool resource [13].

In an environment with constant pressure to provide ever-increasing and costly healthcare services [14–16], a general framework to assess the sustainability of these healthcare systems is desperately needed. The *a priori* core assumptions that must be agreed upon before developing such a sustainability framework are that these healthcare systems are complex with multiple components that interact in both linear and non-linear ways, and two, there will never be a single governance solution that applies to all systems [17]. The common pool resource model provides an ideal description of the two defining characteristics of our publicly funded healthcare system; it is difficult to fence off access to resource use, and use by someone reduces access by another user [13]. In Ostrom’s model of human resource use, she describes a complex socio-ecologic system composed of four core sub-systems; resource systems, resource units, governance systems and users [13]. Each of these is composed of other relevant variables important for the analysis of system sustainability. For example, across many different ecological systems, the importance of the resource to users and users’ deep understanding of the system is critical to users’ involvement in self-governance and sustainability of the resource.

While Ostrom developed this system to explain the patterns of human use of ecological resources, the analogy to our publicly funded healthcare system could provide a framework against which hypotheses about governance could be tested. In this model, the greatest threat to the sustainability of common pool resources is the failure to establish rules and norms for resource management [18]. These rules and norms are commonly referred to as governance [19]. In a recent systematic review of healthcare system governance frameworks [19], only a single study was found that applied the common pool resource model to analyze primary healthcare governance in low and middle-income countries [20]. In their governance framework, Abimbola et al. conceptualized governance as consisting of multiple levels: operational governance (local users and their healthcare providers), collective governance (community coalitions), and constitutional governance (governments at different levels and other distant but influential actors) [20].

The operational level of healthcare system governance in Abimbola et al. [20] refers to the day-to-day rules and norms used by local users and healthcare providers to make and implement practical decisions. Both users and healthcare providers may belong to more than one level of governance, depending on whether they act alone or collectively. Rules may be both formal and informal. Depending on the context, many factors may influence governance. For example, do users have different attitudes and perceptions about their roles in governance? What are the values, beliefs and cultures that influence the decisions that local users make about their role in the sustainability of the healthcare system?

If local users and healthcare providers establish rules and norms and there is a breach of these rules and norms, the question of patient accountability becomes relevant. To date, there is a substantial amount of literature that focuses on accountability from the perspective of the payer and provider in healthcare [21]. However, there is less known about accountability by patients. Currently, there is no definition in the literature for “patient accountability.” Editorials and commentaries on patient accountability often describe two types of patient accountability. The first type of patient accountability refers to the *patient’s responsibility for management of their healthcare condition(s)*. The underlying assumptions for this responsibility are that the patient is well informed by their healthcare practitioners on managing their health condition(s) and is physically and mentally capable of managing their health condition(s). This can include responsibility for a patient’s healthcare before becoming ill, and illness management after diagnosis and treatment are provided. The second type of patient accountability refers to the *patient’s responsibility for the use of the healthcare system*, such as the responsibility to use public resources for healthcare in a manner that allows for long-term affordability, sustainability, equity and availability to all [22–24] (e.g., utilization of low-value services for end-of-life care, unnecessary diagnostic tests, and referrals; use of duplicate services and time and resources spent on frivolous complaints against organizations and providers).

There are various definitions of “accountability,” however, the simple definition is “answerability,” which is the obligation to answer questions regarding decisions and/or actions [25–27]. This definition can be extended to be inclusive of the terms of sanctions. To clearly understand accountability in any context, it is crucial to answer the questions of: ‘for what, by whom, to whom and how’ [26]. A meta-narrative review on public accountability identifies four paradigms for accountability [21]. The Institutionalist paradigm is when formal procedures and instruments and social norms in organizations and institutions are used to improve accountability. Enforcement is through verifying compliance with procedures, rules, laws, and policies. Rights-based is when accountability rests on individual human rights or entitlements. Citizens delegate power and authority to government/public sector institutions, which in turn are accountable for the realization of citizens’ rights and entitlements. For individual choice, accountability is from the client’s perspective and relies on competitive market behaviour to explain accountability relationships. Individuals, organizations, and institutions are connected by multiple accountability relationships. Actors can be account holders for some actions and account for others at the same time. The meaning of accountability is socially constructed and dependent on a given context.

Brinkerhoff et al. [25] identify three dimensions of accountability. *Financial accountability* is tracking and reporting on allocation, disbursement, and utilization of financial resources through auditing, budgeting, and accounting. *Performance accountability* is the demonstration of performance based on agreed-upon performance targets [25]. *Political/democratic accountability* is the institutions, procedures, and mechanisms that ensure that government delivers on electoral promises, fulfills the public trust, represents citizens’ interests and responds to societal needs and concerns [25].

Financial, performance and political accountability ensure that resources are used, and authority is exercised according to legal procedures, professional standards, and societal values. This can translate into payer accountability (where resources go); patient accountability (how resources are used); and clinical accountability by providers (how resources are used and how services are delivered, individually or in teams) [26]. Accountability is multi-directional and involves multiple combinations of actors, including providers (public and private), patients/service recipients, payers (including insurers and the legislative and executive branches of government) and regulators (governmental, professional) [26]. There are different approaches for enforcing accountability that vary from: penalties embodied in laws and regulations, the use of financial incentives/expenditures, professional codes of conduct, market mechanisms of supply and demand, public exposure or negative publicity [25] (e.g., the “naming” and “shaming” of patients as consumers who have acted inappropriately [28], the provision of performance information to payers and the public and professionalism/stewardship [26]. The degree of accountability that is valuable is debated. O’Neil [29] has argued that more accountability is not always better, and processes of holding to account can impose high costs without securing substantial benefits. In the worst situations, they can damage the performance of the very first order tasks for which they supposedly improve accountability. For this reason, profound and effective accountability must be founded on good governance and less on control, on obligations to tell the truth, and seeking intelligent accountability.

In an environment of demographic changes and fiscal constraints, there is a need to examine the approaches that can result in the more responsible use of services by consumers of healthcare. To date, no analysis has been done to explore the frameworks and definitions for patient accountability. This study aims to provide an overview of frameworks and analyze the definitions of patient accountability to identify themes and potential gaps in the literature.

## Materials and methods

A scoping review is a type of literature review focusing on a topic but does not evaluate the quality of the research. This scoping review was conducted using the five-stage approach by Arksey and O’Malley: (1) developing research questions, (2) identifying relevant studies, (3) study selection, (4) charting data, (5) reporting the results [30, 31]. The optional 6^th^ stage of consultation was not adhered to as we did not have a clear purpose for the consultation nor the financial resources to do so. Table 1 details the process that was followed for each stage of the study.

**Table 1:** Methodology guiding the scoping review.

Framework Stage	Study Details
Stage 1: Determining the Research Questions	The following research questions guided the scoping review: 1. How is patient accountability defined in healthcare? What are patients accountable for? Who are they accountable to? What is the role of the patient? 2. What theories are used to define patient accountability? 3. What are the main approaches to addressing patient accountability (i.e., how are patients held accountable?)?
Stage 2: Identifying Relevant Studies	Fifteen bibliographic databases were searched until July 25^th^, 2021. This included: MEDLINE (EBSCOhost), EMBASE (OVID), CINAHL (EBSCOhost), PsycINFO (Proquest), SPORTDiscus (EBSCOhost), Allied and Complementary Medicine Database, Web of Science, HealthSTAR, Scopus, ABI/INFORM Global, Cochrane Library, ERIC (Proquest), International Bibliography of the Social Sciences, Sociological Abstracts, Worldwide Political Science Abstracts and International Political Science Abstracts. This was complemented with searches in Google Scholar. No time limitation was used in the search. To develop the search strategy, four experts (as identified by the core team and the literature) were engaged to draw on their perspective and knowledge of key articles related to concepts consistent with the ideas of patient accountability. The reference lists of identified articles were reviewed to identify additional relevant articles. The abstracts and (if necessary) full articles for these citations were read to determine if the article provided a definition, frameworks, or any other information that could be used to describe the concept. Identified articles were used to develop the search and review criteria by the research team in consultation with a medical librarian with extensive experience conducting scoping reviews. The final search strategy for MEDLINE can be found in S1 File. The search terms included a combination of subject headings and free-text terms. This combination of keywords varied as per the different indexing schemes used in each of the databases. Final search results were exported into RefWorks, and duplicates were removed. In addition, to the systematic database search, a manual search was done of reference lists. We also conducted searches in Google Scholar.
Stage 3: Study Selection	Study selection was based on our inclusion and exclusion criteria. Articles were considered for *inclusion* if they focused on the accountability of patients. Due to the nature of the topic and to prevent missing any relevant publications, articles were not excluded based on study design. Articles were only *excluded* if they were not available in English, were commentaries or editorials, did not have abstract or full text available, or had missing information. Initial screening of articles was based on the review of titles and abstracts. The remaining full-text articles were further screened based on the inclusion and exclusion criteria. Four independent reviewers screened the titles and abstracts (at the first stage of screening) (MA, KMK, AS, GD) and full-text articles (at the second stage for inclusion or exclusion of the articles) (MA, KMK, RG, AS) using a predefined charting form. Any disagreements were resolved with the guidance of senior authors on the paper (see Fig 1 for a PRISMA Flow Diagram of article selection).
Stage 4: Charting the Data	Variables extracted for data charting from the selected studies included: author(s), year of publication, study design, article type, study objective, definitions, theoretical approaches/concepts, what patients are accountable for, whom patients are accountable to, and conclusions and recommendations (Refer to S2 File). Data charting was done in an Excel file. Themes emerged inductively from included articles to provide an account of available research. As such, data were also extracted for: arguments for and against patient accountability.
Stage 5: Reporting the Results	The results were reported using thematic analysis. The thematic analysis involved qualitative content analysis, such that themes were identified according to the research questions.

**Fig 1:**
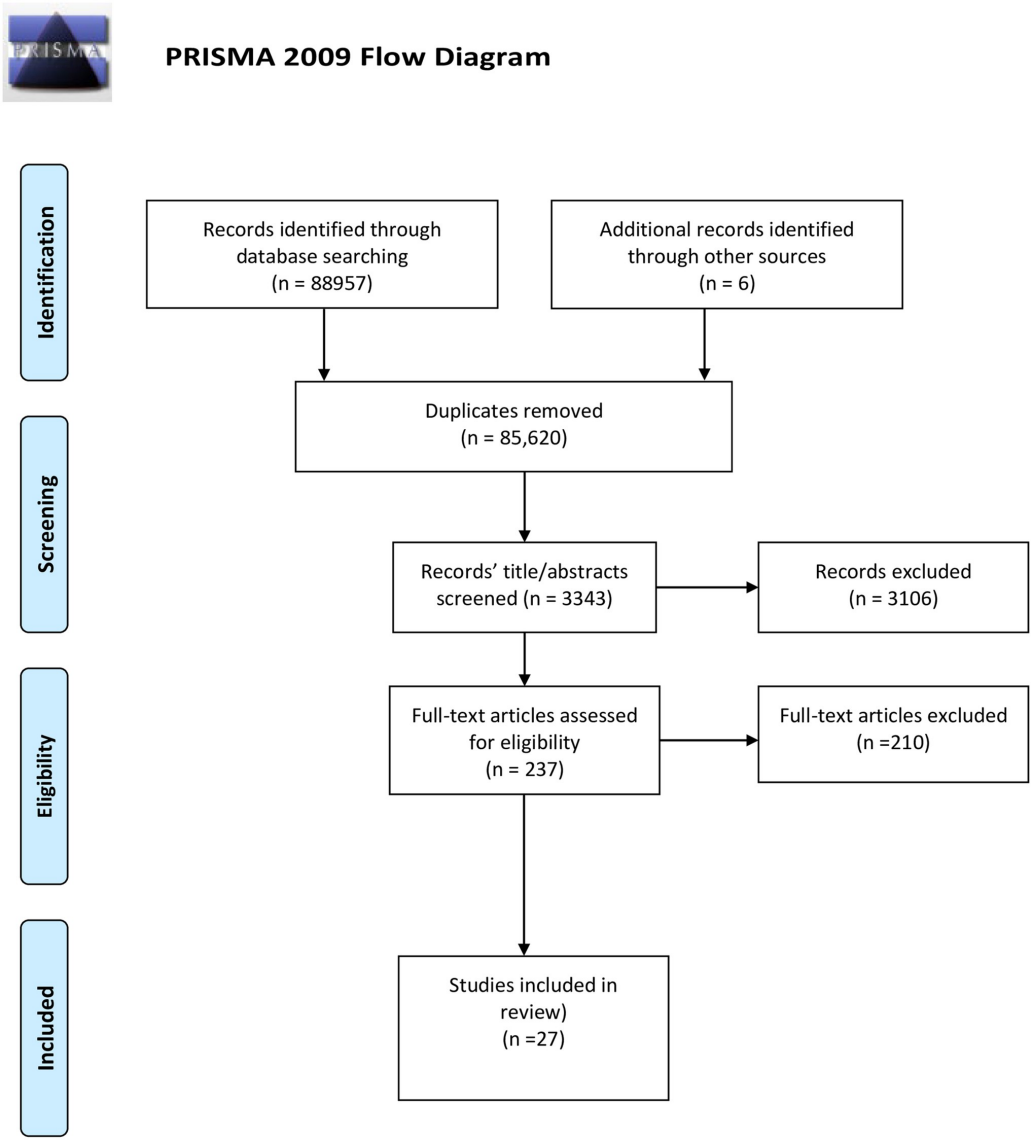
Patient accountability flow. *From*: Moher D, Liberati A, Tetzlaff J, Altman DG, The PRISMA Group (2009). *P*referred *R*eporting *I*terns for *S*ystematic Reviews and *M*eta-*A*nalyses: The PRISMA Statement. PLoS Med 6(7): e1000097. doi: 10.1371/journal.pmed1000097 **For more information, visit** www.prisma-statement.org.

## Results

The Preferred Reporting Items for Systematic Reviews and Meta-Analyses (PRISMA-ScR) checklist for scoping reviews was used to report on the results [32]. (S3 File). 27 studies were included in the review (Fig 1).

### Terms and definitions

The term ‘accountability’ [33, 34], and ‘patient accountability’ was rarely used. The most used terms include the word ‘responsibility’ [35–40]. Assessment of the definitions in the literature indicates that many studies focus on what individuals are accountable for and related consequences rather than providing explicit definitions. The term ‘accountability’ was defined in one study as expectations set by a system in which citizens or patients are held morally and legally accountable for their behaviour [33]. ‘Responsibility’ has been defined as the result of social relationships between individuals, which involves fulfilling obligations and duties [37] and distributing society’s goods and burdens [41]. Responsibility takes effect when an autonomous informed individual [37] is capable of understanding and following moral norms and rules [35] and is responsible for their actions and accountable for their conduct, choices and behaviour [35, 37]. Table 2 provides definitions for different types of responsibility.

**Table 2:** Definitions of responsibility.

Type of Responsibility	Definition
Personal responsibility	Individuals’ have control over the factors that shape choices [42]. Individuals are responsible for their own health [43]; individuals are morally responsible for actions [35]; responsibility is determined by choices made in past or future [44].
Individual Responsibility	The individual is personally responsible for life-style related choices [43]. Individuals are morally responsible for their actions [33].
Role Responsibility	A person’s body belongs to oneself, which includes responsibilities toward oneself [45, 46].
Causal responsibility	A person causally contributes to one’s disease [46] through personal behavioural choices or an act that directly contributed to an event [45–47]. Causal responsibility is a condition for moral responsibility [35].
Attributive responsibility	Indicates it is appropriate for a person to be subject to moral appraisal and to assess individual actions for blame or praise [43].
Blame responsibility	An individual is blameworthy for choices [46].
Substantive responsibility	Actions people are required to do for each other and regulate distributive justice [43].
Moral Responsibility	Based on the kind of person one is, how one assesses, chooses and acts, and how one responds to the outcomes of one’s actions and impact on others [39]. Moral responsibility is dependent on causal responsibility [35, 45], which is when individuals are held morally responsible for choices within their control; however, some outcomes may be excusable or justifiable [35]. Moral responsibility implies that a patient deserves the outcome [48].
Social Responsibility	Individuals are responsible for the health of society. Social responsibilities include addressing the determinants of causes of health, obtaining public participation, and using various approaches (legislation, organization change and community development) [47].
Co-responsibility	Responsibility is a dynamic between individuals and not just individually based [38]. Co-responsibility differs from a shared responsibility, as people do not own the same responsibility but instead have different, individual interdependent and more autonomous responsibilities [49].
Patient responsibility	Patient responsibility: A patient is responsible for actions [50] related to their choices and health problems [48], for duties to health care providers [51].
Physician-patient responsibility	A patient is responsible for working with clinicians regarding their treatment goals. The clinician is responsible for obtaining information from patients to foster established goals [48].

### What are patients accountable for? What is the role of the patient?

The majority of studies suggested that it’s appropriate to expect patients to be accountable for their health-related behaviours [33–47, 49, 51–55] whether as a preventative strategy before the onset of disease [34, 37–47, 49, 51–55], as a compliance strategy to minimize the health-related effects of disease [41, 44, 49, 51, 54], or as a responsible utilization strategy to minimize the waste of limited healthcare resources [48, 51, 53–55].

Studies indicated that individuals are accountable for lifestyle choices and risks that could have been avoided [33, 34, 37–39, 41–43, 47, 48, 56], attempting to make the best choices for their health [33, 35, 52, 56] and implementing health promotion and healthy living and safe activities [49, 51]. A study highlighted patient responsibilities, which included being truthful, providing a complete medical history, requesting information or clarification, complying with physician instructions, meeting financial obligations, being cognizant of the costs associated with healthcare, using medical resources judiciously and discussing the end of life decisions and organ donation [54]. Furthermore, patients take responsibility for their health by accepting the assistance of healthcare providers and involving family members in decision-making. This highlights the importance of individualized care or patient-oriented care [49]. Thus, patients should be willing to accept help for their care while also keeping in mind that their health is under their control instead of completely shifting their roles onto their healthcare providers [56].

### Whom are patients accountable to?

Studies indicated that patients have a “moral obligation” to be accountable to themselves [48, 53], the physician/clinician/contract provider/healthcare provider providing their care and the care of others [36, 37, 43–45, 50, 51, 53], and/or to the healthcare system or society [38, 41, 42, 45, 47, 48, 51–55]. Only one paper considered multi-level accountability between all three [53].

### What theories are used to define patient accountability?

The most common theories identified about patient accountability included Eli Feiring’s concept of *forward-looking responsibility* in healthcare [39, 41, 42, 44, 50, 51], which states that what matters morally in the allocation of scarce healthcare resources is not people’s past behaviours but instead their commitment to lifestyles that will increase the benefit from treatment. Backward-looking responsibility [39, 41–44, 52] was also identified, a concept often associated with luck egalitarianism, an influential theory of distributive justice. Luck egalitarians assert that distributions are justified based on voluntary past choices [44, 52]. The rational choice theory was also identified; this theory states that individuals depend on rational calculations to make rational choices that result in outcomes aligned with their own interests [37, 52]. Utilitarian and deontological principles are essential to defining accountability, as these approaches emphasize concepts that encourage patient autonomy, such as informed choices and discussions of risks and benefits [57]. Much of this literature debated the most appropriate approach for patient responsibility for healthcare.

### What arguments support patient accountability?

The debates in favour of patient accountability for some action or behaviour were based on the ability to make rational and responsible choices [33, 34, 36–39, 41–43, 45, 47, 48, 50, 51, 53, 55], costs to society [43, 54], avoiding moral hazard [45, 52], responsibility for scarce resources [44, 51, 54], and the need to enforce behaviour. Debates that argued against patient accountability for behaviour or consequences were based on: whether a behaviour had resulted as a consequence of choice or circumstances such as socioeconomic status, poverty, ignorance or involuntary behaviour [34, 36, 39–41, 43, 44, 46–48, 50, 51, 53]; healthcare is a unique good [41, 58]; inability to make rational choices [55]; prejudice in ascribing responsibility; medical condition such as mental health or degree of autonomy; the ability to comply [37]; outcomes that can result from accountability such as inequality, reducing access to healthcare or impact on relationships with providers [43, 45, 52]; the resource-intensive exercise of determining responsibility [45]; ethical, fairness, compassion and humanitarianism principles to help those in need [39, 41, 42, 47]; infringing on individual rights, freedoms, and autonomy to make lifestyle choices [38, 39], and victim blaming [41].

### What are the main approaches to addressing patient accountability (i.e., how are patients held accountable)?

There are several different mechanisms for enhancing patient accountability including: rewards [42] or financial penalties/sanctions [41], taxes on unwanted behaviour [37, 47, 48, 52], insurance premiums [36, 48]; contracts and written agreements [50, 51]; lower priority on waiting lists [36, 44]; denial of treatment for non-compliance [36, 39]; and consumer-directed health plans [55]. A common criticism of many patient accountability frameworks lies in their unrealistic assumptions that patients are autonomous agents capable of rational, informed, independent, and unbiased decisions about their own health. As a result, many studies reinforced that any future patient accountability frameworks must refrain from being punitive, stigmatizing or further exacerbating the socioeconomic disparities that may be causally related to differences in health-related behaviours and outcomes. These studies acknowledged that a shared model of accountability between patients and their healthcare providers, and between patients and their communities/society were the preferred and fairer models [34, 37, 38, 40, 46–48, 50, 51, 54, 59]. In addition, recognizing and integrating the origins of responsibility, (which include personal maturity, civic duties, respect for autonomy, and relational duties to loved ones and physicians), to these approaches are essential [56].

## Discussion

We sought to describe frameworks on patient accountability. Among the 27 articles reviewed, there was a lack of consensus on how patient accountability is defined and whether it should be defined at all. None of the literature was dedicated to the emergence of biosensor devices that could be used to objectively measure and monitor patient behaviours such as medication adherence, physical activity, or cessation of cigarette and alcohol consumption. Nor was there any mention in the literature of the potential impact precision medicine may have on tailoring treatment to predict the benefits and risks individual patients might experience more definitively. Theoretical debates focused on whether accountability should be assessed for past or future behaviour [39, 41, 42, 44, 50–52]. There was general agreement that patient autonomy about health behaviours and compliance with medical treatment and care plans should be part of a patient accountability framework [38, 39]. However, how and by whom this would be measured, monitored, and enforced was unclear. Healthcare providers were essential and pragmatic mediators of patient autonomy by monitoring patient behaviour and compliance. The literature indicated that patients are accountable to themselves, their healthcare providers and the healthcare system/society as a whole [53, 60]. Scholars have recommended that healthcare systems hold patients accountable by having patients more actively involved in their healthcare decision-making, rather than leaving the decision to physicians [61]. As a result, patients would have the added accountability of making autonomous and well-informed decisions [61].

Several studies identified incentives as potential influencers of patient accountability through either improved access or reduced costs of healthcare services [36, 44]. Patient accountability mechanisms identified included financial penalties, sanctions, taxes on unwanted behaviour, insurance premiums, lower priority on waiting lists, and denial of treatment for non-compliance. However, disincentives were seen as ethically and morally problematic unless patients were fully empowered to affect change. Most authors supported the notion that any patient accountability framework should incorporate consideration of the patients’ ability to affect their own health, whether through environmental, genetic, or socioeconomic influences, and that society must play a role in supporting the determinants of health. Furthermore, the literature did not address the legal, social, and economic costs to implement a patient accountability framework. More research is needed to determine whether these costs would outweigh the benefits of implementation or contribute to a more sustainable healthcare system.

In the healthcare system, there are some examples of patient accountability frameworks that can impede access to life-saving medical treatments [62]. Organ transplantation represents life-saving medical treatments, but due to limited organ donors [62, 63], the number of transplant applicants far exceeds the number of donors across countries [63]. In Canada, before any patient is considered for lung [64] or liver [65] transplantation, they must meet eligibility criteria that are disease-based and demonstrate the absence of absolute contraindications that include both forward and backward-looking behaviours [39, 41, 42, 44, 50]. Patients who are currently or historically non-compliant with medical treatment are excluded from being listed for lung or liver transplantation. Backward-looking non-compliance has been shown to increase the forward-looking post-transplant risk of non-compliance with anti-rejection medications, follow-up appointments, and medical advice, all of which may jeopardize the outcome of transplantation. Patients with substance misuse behaviours that include cigarette smoking or alcohol consumption are also ineligible due to concerns that potential donors might reconsider if they knew their organs might be transplanted in patients who had substance misuse behaviours.

While limited in scope, these examples demonstrate that patient accountability frameworks have been implemented in situations where there are no alternatives to these treatments, and exclusion inevitably leads to negative health outcomes. Even in the most severely limited healthcare resource situations, such as organ transplantation, the justifications provided for impeding access are not supported by the conclusions in the majority of papers in this scoping review. Some of the arguments against such a patient accountability framework include that healthcare is a human right [66], personal consequences should not be severely punitive or life-threatening [46, 50], or that the patient accountability framework is unethical [45]. The lack of public acceptability of such accountability frameworks represents another threat to their implementation. For example, there is a pending Canadian Charter of Rights and Freedom challenge regarding the constitutionality of excluding liver patients from access to transplantation based on lack of abstinence from alcohol ingestion for a period of at least six months before transplantation [67]. In some jurisdictions in Canada, this exclusion has already been reversed due to public pressure and emerging evidence that this behaviour may not be as impactful on transplant outcomes as previously believed, reinforcing the inherent uncertainty in estimating the negative health consequences of patient-modifiable behaviours.

### Strengths and limitations

The strength of this study is the search was conducted in 15 bibliographic databases and three authors reviewed full-text articles independently. The limitations were primarily rooted in the dearth of literature published on patient accountability, with most of the literature focused on the ethical issues of patient accountability. Both biosensor devices and precision medicine depend on patients agreeing to share even more personal health information, a component of patient accountability that was never mentioned in any of the studies included in the review.

## Conclusions

From this review, it appears there is a lack of agreement on what constitutes patient accountability and how it should be implemented. The essential components of any patient accountability framework will require objective tools to measure and monitor patient behaviour and a process for reviewing compliance. Implementation will require a legal framework to administer socially and politically acceptable incentives and disincentives. Less clear is whether any of these efforts to implement a patient accountability framework in the healthcare system will improve patient outcomes or cost-effectiveness. While all the aforementioned components may be necessary to start designing a patient accountability framework, social and political acceptability may represent the most important challenge in this process. Therefore, engaging the public in an honest and open discussion about patient accountability is an essential first step.

## Supporting information

S1 File MEDLINE search. (DOCX)

S2 File Data extraction. (DOCX)

S3 File (DOCX)
