# Supplementary material for: The role of patients in the governance of a sustainable healthcare system: A scoping review
Source: PLoS One. 2022 Jul 13;17(7):e0271122. doi: 10.1371/journal.pone.0271122 (PMC9278783; doi:10.1371/journal.pone.0271122)
Supplement: S1 File — (DOCX) [file pone.0271122.s001.docx]

**Supplemental File 1**: Patient Accountability EBSCO MEDLINE Search Strategy (Literature search performed: Feb. 28, 2018)

1. (patient OR user OR consumer).mp
2. (accountability OR governance OR activation OR duty OR obligation OR responsibility OR participation OR self-management OR self-care OR liability OR answerability OR involvement OR "mediated intervention" OR "shared decision making" OR "health behaviour" OR "health behavior").mp
3. (healthcare OR healthcare OR health-care OR "healthcare system" OR "healthcare services").mp
4. AND/1-3
5. (accountability OR governance OR activation OR duty OR obligation OR responsibility OR participation OR self-management OR self-care OR liability OR answerability OR involvement OR "mediated intervention" OR "shared decision making" OR "health behaviour" OR "health behavior").mp
6. (frameworks OR approaches OR concepts OR ideas).mp
7. (healthcare OR health-care OR "healthcare system" OR "healthcare services").mp
8. AND/1-3
9. OR/4,8
