# Supplementary material for: The role of patients in the governance of a sustainable healthcare system: A scoping review
Source: PLoS One. 2022 Jul 13;17(7):e0271122. doi: 10.1371/journal.pone.0271122 (PMC9278783; doi:10.1371/journal.pone.0271122)
Supplement: S2 File — (DOCX) [file pone.0271122.s002.docx]

| Author, Article Type, Country | Objective | Terminology | Theory/  Concepts | Arguments For | Arguments  Against | What Are Patients Accountable For? | To Whom Are Patients Accountable To? | Conclusion & Recommendations |
| --- | --- | --- | --- | --- | --- | --- | --- | --- |
| Albertsen (2015),  Ideas,  Denmark | To critically examine Feiring’s concept of forward-looking theory | Personal responsibility | Forward looking personal responsibility  Luck egalitarianism | We need to be attentive to how much good resources will do for the person they are allocated to | Difficult to assess whether something has happened as a consequence of choice or circumstances | Lifestyle changes and commitment to contract | Contract Provider | Circumstances may affect the choices after commitment to lifestyle  Provide priority for individuals willing to commit to lifestyle change |
| Andersen (2013),  Idea,  Denmark | To examine personal responsibility and luck egalitarian theory with respect to social inequality in healthcare | Individual Responsibility  Attributive responsibility | Luck egalitarian  Egalitarianism | Costs associated with imprudent behavior should fairly be passed on to the imprudent | People that belong to a group that is more prone to a certain risk factor shouldn’t not be held responsible | Their lifestyle-related diseases | Physician | We must work to provide everyone with equal access to healthcare in order to reduce health inequality  Attributive responsibility should be assigned to individual actions, rather than substantive responsibility |
| Andersen (2015),  Ideas,  Denmark | To examine whether and how  luck egalitarianism can justify universal  health care | Personal Responsibility | Luck Egalitarianism  Rawls’ theory of justice. Three principles:(1) equal liberties, (2) fair equality of opportunities, and (3) the difference principle. | There is reason to avoid or at least reduce moral hazard | Those impacted tend to be from lower socioeconomic status | Their health | Healthcare system | Efficiency-based reasons for cost responsibilities are not convincing. A balance between efficiency and equality is required  Regulate individual behavior via consumer taxes. |
| Axtell-Thompson (2005), Ideas, USA | To examine the debates related to consumer directed health care (CDHC) | Consumer directed health care | Consumer Sovereignty – consumers can make informed decisions for which the criteria are capability, information and choice | Patients with sufficient information can participate in rationing and cost-control to make choices | There is no evidence it results in cost control  Consumers may commit to health plans choices based on current preferences and circumstances without recognition of changing health needs or changing circumstances | Health related behaviors  Appropriate use of resources | Society | Consumer directed health care plans structured in a way to ensure consumer choice and responsibility for health care do not exacerbate current disparities in health care |
| Baeroe (2015), Analytic Paper, Norway | To use an analytic approach to examine different ways of holding people responsible for healthcare-related choices | Risk-sharing view | Responsibility can be differentiated according to when in a lifecycle it is ascribed | Patients are responsible for health risk that could have been avoided | Public obligation to help  people who need medical intervention and  avoid risk  It can be hard to attribute lifestyle to a disease | Their health and lifestyle | NR | Patients should be held  responsible for failure to attend required programs if free of charge and easily accessible |
| [Björk](https://www.tandfonline.com/author/Bj%C3%B6rk%2C+Joar) (2021), Qualitative, Sweden | To analyse and describe general practitioners’ perceptions of the notion of a ‘personal responsibility for health’ | Personal Responsibility: responsibility coming from within yourself; from your relationships to specific others; and/or from your relationship with the generalized other | Human dignity principle: prohibits taking previous behaviour into consideration when setting priorities  Principle of responsibility: patients whose imprudent behaviour has contributed to the establishment of their disease should be down prioritized for treatment in comparison with patients with no such history of imprudence in health | Responsibility for one’s health is included in one’s general responsibilities for one’s welfare  The responsibility for your health arises as a consequence of your bonds to those close to you  Whenever you and your GP reach a treatment agreement, you become responsible for that facet of your health  The link between responsibility and freedom provides an obligation for others to treat you as responsible for your actions  If one doesn’t take care of oneself, it will be expensive to everybody, including society, healthcare and the taxpayer | There is no responsibility for health in the absence of close relationships.  Adherence and responsibility for health is part of a shared undertaking rather than a feature of the individual patient. | Owning their health problem, not offloading all responsibility onto the GP, taking active measures to keep and improve health and accepting help in health. | Oneself, others and the physician. | The physician was described as playing a key role in shaping and defining the patient’s responsibilities for his/her health |
| Cappelen (2005), Ideas, Norway | To examine debates related to responsibility in health care | Personal responsibility | Forward looking and backward looking | Arguments related to Forward and Backward-Looking Responsibility | Normative Objections | Responsible for lifestyle choices | Healthcare system | Reward wanted behavior or tax unwanted behavior rather than the consequences of the action  If governments or insurance plans cover in full screening programs such as mammography, smoking cessation programs, and vaccinations, as well as testing and treatment for sexually transmitted diseases appropriate behavior can be encouraged |
| Carvalho (2021), Qualitative, Brazil | To analyze health professionals’ perception about the meaning and practice of patient involvement in care safety in Primary Health Care | Co-responsibility  Patient-centered care  Patient empowerment |  | Patient need to understand that they are the leaders of their health-disease process and need to be active and autonomous individuals  Much of the percentage of the problem is solved by the patients themselves, taking their own care  Patients must perceive and act on the risks involved in their health-disease process | As there is little time for consultation, sometimes patients leave without knowing exactly how their medical should be used  There is lack of training for physicians for patient involvement in care  There is lack of patient literacy  Some patients lack hearing capacity which affects communication and affects their adherence to treatment  Some patients lack family participation in care, which has been shown to be essential for helping cope with the health-disease process | Their security; for the use of medications, in the warning signs and understanding when it is getting worse  Commitment to following the physician’s guidelines, so that the patient maintains their safety  Patient involvement | Clinician | The factors involved in patient involvement in care safety were related to aspects of patients, health professionals and the health organization itself |
| Civaner (2008), Analytic,  Turkey | To examine types of patient responsibilities and their appropriateness | Patient Responsibility | NR | Patients are responsible for the consequences to their health if they refuse treatment  or do not follow practitioners’ instructions | Individuals  have limited or no control over many factors that influence heath | Responsible for following rules and health and society | Patients, health care workers and society | Right to health is the right to required health services and are accessible for everyone regardless of lifestyle. Sanctions that limit access are unacceptable |
| Devisch (2012), Ideas,  Belgium | To examine the use personal responsibility in healthcare and the concept of co-responsibility | Co-responsibility | NR | NR | It logical to hold the individual personally responsible for making  unhealthy lifestyle choices | Responsible for risky behavior | Society | Using co-responsibility as a perceptual lens rather than individual responsibility  A broader framework for handling questions of responsibility in health care is needed |
| Feiring (2008),  Ideas,  Norway | To determine whether responsibility should be used as criteria for healthcare rationing | Responsibility | Luck egalitarianism  Distributive justice  Backward-  looking personal responsibility  Forward looking responsibility | People should be responsible for healthy lifestyle choices | Health care is a special good and should not be allocated to ensure people get what they morally deserve | Responsible for lifestyle  choices  Not making preventative healthcare choices | Healthcare system | Forward looking responsibility should be endorsed rather than backward-looking responsibility. Patients should be liable for their actions in future  To enforce compliance patients with medical conditions are required to sign contracts |
| Freison (2018),  Ideas,  USA | To examine the concept of personal responsibility in the literature | Causal responsibility | NR | We have obligations towards others who have a claim to medical resources | Extensive time and resources are required to assess each individual’s responsibility | Their actions | Society | Moral responsibility for poor health outcomes is explained by social biases rather than many socially acceptable behaviors  Changing individual attitudes,  building experiences of self-efficacy and altering societal norm, motivate positive behavior change, rather than punishment |
| Gauthier (2005), Ideas, USA | To examine moral responsibility as an important element of health care decision making | Moral responsibility | Ethical concepts: autonomy, beneficence,  nonmaleficence, and justice | Patients have obligations within the health care system | NR | Being truthful, providing a complete  medical history, requesting information or clarification, complying  with physician instructions, meeting financial obligations, being cognizant  of the costs associated with health care, using medical  resources judiciously and discussing end of life decisions and organ donation | To others and to the community | The virtue of moral responsibility justifies patient responsibilities  Patient participation in making health care decision, fostered by physician |
| Guttman (2001),  Ideas,  USA | To outline a framework that contextualizes consequences of campaign appeals to personal responsibility | Individual Responsibility | Causality: personal responsibility inherently assumes causal connections between people’s deeds and health outcomes | Those who behave irresponsibly should be held responsible | Lack of clarity on whether individuals have control over practices that lead to health outcomes | Their health | Not relevant (NR) | Ethical implications of holding patients accountable must be understood  Healthcare workers should concentrate on the issue instead of the individual |
| Hansson (2018), Ideas, Sweden | To examine the concept of responsibility and the types that should be avoided in clinical practice | Role responsibility | NR | NR | Physicians cannot know when a patient has failed to do their best | Their lifestyle | NR | Physicians should encourage task responsibility by emphasizing the possibility of success  Provide positive reinforcement for patients trying to comply with physician instructions |
| Iltis (2005),  Ideas,  USA | To examine the concept and debates in the literature on patient responsibilities | Patient responsibility | Debates focus on ethical concepts: the principles of autonomy, beneficence,  nonmaleficence, and justice | Failure to adhere to treatment places others at risk  Moral responsibility to others | Lack of consensus to the assignment of patient responsibility due to physical or emotional vulnerability, illiteracy, financial hardship | Adherence to treatment, truth-telling, preventing harm to others, taking care of health and accountability for unhealthy actions, actively promoting healthy actions, health care financing | Physicians, Society | There are advantages and disadvantages to holding patients accountable  Help patients with making better choices and outcomes  available in the future |
| Jerofke-Owen (2018), Qualitative, USA | To examine patients’ experiences and preferences for engaging in their healthcare while hospitalised | Patient engagement |  |  | Preferences for engagement are not assessed while hospitalised, leading to patient role confusion and frustration  The majority of patients stated that there were few decisions to be made.  a one-sized fits all approach to patient engagement should not be used in practice  In a study, nurse receptiveness to patient participation and questioning was limited, with nurses often ignoring patients and talking amongst themselves  Patient engagement can be influenced by the unfamiliarity of the place, creating anxiety and fear  Patients may encounter unfamiliar faces, making them uncomfortable to ask questions  Patients’ engagement in care is less likely when nurses seem task-orientated and busy  Patients may be hesitant to ask questions due to the embarrassing nature of the topic  Some patients don’t ask questions because during rounds, providers talk at a high level that is difficult to understand, some find it intimidating having so many people in the room and others just ‘go with it’ because they know the provider is busy and wants to get out of there | partnerships and strategies should be established with patients that can be used to engage patients in processes of decision making, goal-setting, treatment planning and self-care through a model of shared accountability.  Being involved in their self-care activities such as ambulating in the hallways, making good food choices and taking their medications as directed  Sharing subjective information that the provider might not otherwise know | Healthcare system | Engagement is a dual responsibility of both providers and patients |
| Kelley (2005), Ideas, USA | To examine the nature and extent of patient responsibility | Patient responsibility | Forward-looking approach | A medical culture with a more moderate notion of patient responsibility is preferable to any system of enforced patient responsibilities | Adopting a model of shared responsibility could undermine compassion for the vulnerable  Individuals  cannot chose their genes. | NR | Physician | Argues in favor of forward-looking responsibility  Encourage patients to be aware of the consequences of their actions |
| Kjellström (2011), Qualitative, Sweden | To reason about older people’s responsibilities over health and as patients | Responsibility has different meanings at different stages of performance, so the term can be used in several ways | NR | NR | Health promotions for older persons  require interventions to address the individual, the physical environment, and  social environments  Individuals may not have the capabilities to comprehend or take responsibility | Responsibility for health | NR | There is variation in how the elderly reason about responsibility for health and their responsibility as a patient, ranging from no to some reasoning  To be better able to adopt responsibility for their health, patients shall be supported |
| Lakeman (2016), Ideas, Australia | To examine responsibility in the context of mental health, drug and alcohol abuse | Responsibility | Autonomy: the right to self-determination and to make  choices with understanding without controlling influences | Intoxication is invoked as an  excuse for many to evade responsibility | A person who is ill (especially in mental health cases) cannot be held morally responsible | Their choices and behaviour | Clinician | Clinicians are encouraged to consider the context-bound nature of personal responsibility and how attributions of personal responsibility may conflict with policy and their own professional responsibilities to intervene to protect others  Health professionals need to maintain safety and their own responsibilities |
| McDonald (2007), Qualitative, UK | To explore ways in which participants engage in identity work in the context of decisions about healthcare preferences and assesses the extent to which this is compatible with the identities promoted in government policy (consumerism) | NR | Ethical Consumer: self-definition and self-constraint  by which individuals train themselves to become ethical persons | NR | In the context of healthcare, conceptualizing patients as consumers is inappropriate | Government policies emphasize responsibility for health | Healthcare system | The ethical consumer appears to accept responsibility for self-regulation with regard to their consumption of health services |
| Michailakis (2010),  Ideas,  Sweden | To examine the communicative structures that make the shift to individual  responsibility | NR | NR | When a sick person does not meet obligations properly, the provider has the right to deny benefits | Those who act involuntarily, through ignorance, cannot be held account notable | Their lifestyle | Physician | Medical and political observations of illness should be separated  Poor choice makers should be penalized |
| Minkler (1999), Philosophical, USA | To examine personal and social responsibility for health | Personal responsibility | Dworkin’s typology of the several alternative meanings of responsibility in the debate over health promotion and personal responsibility for health | Men who followed more personal health habits had lower mortality than those who followed less | Poverty itself is widely accepted as among the most significant  risk factors for illness and premature death | Their health | Healthcare system | A balance between social and personal responsibility is optimal  Interventions focusing on providing knowledge and  skills for changing unhealthy behaviors |
| Olsen (2000)  Qualitative,  USA | To identify where responsibilities lies for patient healthcare | Patient responsibility:  an individual's actions are their own responsibility and that they should be held morally and legally responsible for the outcomes of those actions  -Physician-patient responsibility:  assuring safety and the best possible outcomes for the patient | Moral responsibility carries with it an implication that the patient deserves the outcome, which has negative healthcare implications  Optimum healthcare is not achieved through defining  the responsibilities of the involved parties  (patients, clinicians and society) but through the  harmonious interaction of all three. | Patients should be held causally responsible for their actions | All too often, the determination of responsibility is about assigning  blame and determining liability | Not Reported (NR) | Physician | Patients should be held responsible casually but be careful when trying to ascribe moral responsibility |
| Resnik (2014),  Ideas,  USA | To examine the impact of advances in genetics on the responsibility of health | Personal responsibility | Kantian ethics: emphasis on duties such as promise keeping and truth telling obligates adherence to agreed-upon treatments | People shouldn’t be held completely responsible to taking actions based on their genetic predisposition | Policies that discriminate individuals based on their genetic susceptibility | Taking effective  action in response to genetic risk | NR | It is possible for individuals to minimize health risk related to their genetic susceptibilities  Written agreements allow patients and physicians to communicate openly and honestly with clear expectations |
| Snelling (2012), Ideas, UK | To examine the concept of responsibility in the nursing | Accountability: a person is responsible if it is fitting  that he/she gives an account for her behavior | The notions of autonomy and responsibility are mutually related | Holding patients accountable is advantageous because it focuses on the agent and what he has done, regardless of the reaction of others | If determinism is true, we cannot be morally responsible for the things we do | Responsibility for health | NR | Responsibility for health is ambiguous and hence needs to be clarified  Therapeutic relationship can incorporate positive reinforcement |
| Vearrier (2021), Analytic, USA | To introduce the model of Utilitarian Priciplism as a framework for crisis healthcare ethics |  | Principlism is guided by four principles of autonomy, nonmaleficence, beneficence and justice  Deontological Principlism deals with the non-crisis ethical framework that guides modern medicine  Utilitarian Principlism deals with a greater focus on promoting the health of communities and populations | Social distancers describe a moral obligation to protect others from disease even if their personal benefits are secondary | Non-social distancers seem social distancing to be unnecessary limitations on personal liberties  There is a historical context of distrust of the healthcare system. In the past, vulnerable populations have been abused in the name of the public good | Following the isolation and quarantine regulations imposed by CDC  Following social distancing | For the protection of the health of the public | Improving the health of populations during the COVID-19 pandemic may require a shift from deontological principlism toward a more utilitarian principlism perspective, maintaining an emphasis on justice nd the promotion of health within its social context |
